# Pseudohyperglycemia Secondary to High-Dose Intravenous Vitamin C Managed as Diabetic Ketoacidosis: An Endocrinological Catastrophe

**DOI:** 10.1016/j.aace.2020.11.030

**Published:** 2020-11-28

**Authors:** Israel B. Orija, Syeda Hira Zahid

**Affiliations:** Department of Internal Medicine, Wellstar Atlanta Medical Center, Atlanta, Georgia

**Keywords:** pseudohyperglycemia, hypoglycemia, point-of-care blood glucose, IV vitamin C, AE, adverse events, BG, blood glucose, CT, computed tomography, D5W, dextrose water, DKA, diabetic ketoacidosis, FDA, Food and Drug Administration, GDH-PQQ, glucose dehydrogenase pyrroloquinoline quinone, ICU, intensive care unit, IV, intravenous, POC, point-of-care

## Abstract

**Objective:**

To create awareness among health care professionals and nurses regarding interference with point-of-care (POC) blood glucose (BG) meter by high-dose intravenous vitamin C and other potential substances. We report a case that probably resulted in the death of a patient from an erroneous interpretation of POC-BG readings due to interference from high-dose vitamin C.

**Methods:**

Retrospective case review

**Results:**

Our patient was admitted following a syncopal episode associated with an acute non-ST elevation myocardial infarction. She was found to have significant hyperglycemia with blood glucose >600 mg/dL on POC testing, associated with moderate ketoacidosis. She was treated with intravenous insulin as a case of diabetic ketoacidosis (DKA). She developed severe hypoglycemia, which was confirmed on a venous BG, and her condition was complicated by an apparent stroke-like state. The patient deteriorated and subsequently died. We found no report of vitamin C causing apparent DKA, as seen in our case.

**Conclusion:**

POC-BG monitoring is very commonly used in intensive care unit settings to monitor BG as they are minimally invasive, convenient, and quick. However, physicians and nurses need to be aware that certain substances can interfere with and alter POC-BG levels, leading to incorrect diagnosis of pseudohyperglycemia or pseudohypoglycemia. This may potentially lead to catastrophic consequences and result in increased morbidity and mortality in intensive care unit settings. The Food and Drug Administration advises against the use of POC-BG meters in critical settings, and they should never be used to diagnose DKA.

## Introduction

Physicians and other health care associates taking care of hospitalized patients should be aware of the limitations of point-of-care (POC) glucometers in the management of patients with suspected hyperglycemia. We report a case of pseudohyperglycemia diagnosed and treated as diabetic ketoacidosis (DKA), due to interference of high dose intravenous (IV) vitamin C with POC blood glucose (BG) meter. Out patient eventually died, and hypoglycemia likely contributed to the death of the patient.

## Case Report

A 71-year-old African American woman with a past medical history of uterine cancer and no medical history of diabetes mellitus presented after a ground-level fall and a syncopal episode. She was initially admitted to the trauma service but transferred to the internal medicine service after being cleared by trauma. Her electrocardiogram showed significant ST depression, and laboratory investigations revealed markedly elevated troponin (13.67 ng/mL) as well as acute kidney injury. She was admitted with a diagnosis of non-ST segment elevation myocardial infarction, but an urgent cardiac catheterization revealed normal coronaries with an ejection fraction of 70%. Her initial venous BG was noted to be 128 mg/dL, while a bedside POC BG, when checked around the same time, was 453 mg/dL. Her POC-BG the following day was reported to be consistently higher than 500 and 600 mg/dL. Her beta hydroxybutyrate (BHB) level was elevated (>13.5 mg/dL) with high anion gap metabolic acidosis (anion gap of 40). She was presumed to be in DKA and was started on the DKA protocol with insulin infusion. Two hours after starting the DKA protocol, a “code stroke” was called on the patient with reports of left-sided facial paralysis, right hemiparesis, and decreased responsiveness. Urgent brain computed tomography (CT) did not reveal any acute changes, and CT angiogram showed normal perfusion. While in CT, she developed ventricular fibrillation arrest, for which she received cardiopulmonary resuscitation with return of spontaneous circulation. She was later intubated for airway protection. She developed cardiogenic shock following cardiac arrest, which required multiple pressor support. She was also started on continuous renal replacement therapy for acute kidney injury but with very minimal ultrafiltrate retrieval due to hypotension. While she remained on the DKA protocol for 2 hours, her venous BG was consistently and persistently low with readings ranging between 16 and 34 mg/dL, while simultaneous POC-BG reading were consistently high at 500 to 600 mg/dL. Insulin infusion was stopped after the discrepancy was noted but dextrose water (D5W) was not started immediately even though venous glucose was reported to be low. The insulin infusion was withheld for 6 hours after the discrepancy was noted but was restarted after 1 of the POC-BG read of >600 mg/dL. An endocrine consult was requested the next day, the insulin drip was discontinued permanently, and continuous D5W was started at this point. Unfortunately, the patient had sustained prolonged hypoglycemia for about 18 hours before D5W was started (see Table).

**Table:** Comparison of POC, Venous Blood Glucose, pH, Anion Gap, and Serum Bicarbonate

Date and time	Point-of-careblood glucose (mg/dL)	Venous blood glucose (mg/dL)	pH	Anion gap	Serum bicarbonate (mEq/L)
4/18/20					
12:21 am	453	128	7.37	40	19
8:30 am	534				
10:00 am	>600				
Insulin infusion started on 4/18/20 at 10:00 am					
11:00 am	>600				
12:36 pm	497	29	7.16	32	18
Insulin infusion stopped on 4/18/20 at 12:36 pm after reported venous BG of 29 but D5W not started					
2:00 pm	>600				
7:30 pm	430	18	7.20	40	13
8:00 pm	>600				
Insulin infusion restarted on 4/18/20 at 8 pm after reported POC-BG >600					
9:30 pm	437				
11:00 pm	483				
4/19/20					
1:33 am	425	16	7.23	25	19
3:00 am	356	27			
5:00 am	188		7.37	18	18
10:00 am	257				
11:23 am	241	34	7.31	15	20
1:49 pm	190	146			21

Insulin infusion stopped on 4/19/20 at 6 am, endocrine consult placed, and patient started on D5W

Abbreviations: BG = blood glucose; D5W = dextrose water; POC = point-of-care.

Surprisingly her HbA1C remained normal 5.1% (32 mmol/mol). Brain MRI brain was suggestive of hypoxemic ischemic encephalopathy, and unfortunately, she continued to deteriorate neurologically, with minimal purposeful response. She coded again a few days later with pulseless bradyarrhythmia. Cardiopulmonary resuscitation was initiated but resuscitation was called off upon family request, and the patient was pronounced deceased after a complicated 8-day hospital course.

The strong discrepancy between venous and POC-BG values led to a suspicion of interference with the POC glucose meter. We performed a retrospective analysis of the case with a thorough chart review and discovered that patient had been on IV high-dose vitamin C infusion as a natural remedy for her uterine cancer. This was confirmed via a telephone conversation with her son. She received her last dose one day prior to presentation at our hospital. He also reported that the patient had been losing a lot of weight recently with poor oral intake and progressive weakness.

Indeed, we realized that this was an instance of a mismanaged case, and we alerted our hospital administration about changing POC-BG meters that were being used in our institution. Unfortunately, as of now, we do not have any alternate POC-BG meters in the hospital. We presented this case as a morbidity and mortality review with our faculty, residents, and hospital administrators and hope to be able to introduce safer and more reliable POC-BG meters in the future. We are also planning to design a questionnaire for asking patients about their recent use of medications that can interfere with POC-BG meters.

## Discussion

We describe a case of pseudohyperglycemia managed as DKA due to interference of high-dose IV vitamin C with POC-BG meter readouts. This case was made more challenging because the patient also had elevated BHB and high anion gap acidosis, which were due to starvation ketoacidosis rather than DKA. Nonetheless, the clues that went against DKA were normal HbA1C, no history of diabetes mellitus, and no insulin requirement after 48 hours of presentation.

High-dose IV vitamin C has been reported to have antioxidant properties; hence, it is used in cancer patients who opt for “natural remedies” for their malignancy rather than an aggressive approach.^1^^,^^2^ It is also commonly used as an adjunct during resuscitation in burn and septic patients due to its anti-inflammatory properties. With the current pandemic of COVID-19 and ever-changing treatment modalities, the role of vitamin C is being explored and can be expected to expand broadly. Despite the widespread use of vitamin C, there is no overwhelming evidence supporting its use. In the CITRIS-ALI trial published in January 2020 that was conducted in patients with sepsis and acute respiratory distress syndome, a 96-hour infusion of vitamin C, compared to placebo, did not significantly improve organ dysfunction scores or alter markers of inflammation in vascular injuries.^3^ On the other hand, 1 potential pitfall of high-dose vitamin C infusion is its interference with POC-BG measurement.^1^^,^^2^^,^^4^

In the USA, approximately 4800 POC-BG tests are performed by nursing staff per month in intensive care unit (ICU) settings, 97% of which come from capillary blood samples. POC glucose meter technology provides a rapid and inexpensive BG result using minimal volume of blood, making this method the most convenient. But this method is less accurate than laboratory (lab) glucose measurements, and whenever a discrepancy arises between the 2, the laboratory sample should be considered more accurate. Measuring POC-BG in the ICU is becoming increasingly controversial and the US Food and Drug Administration (FDA) advises against its use in critical settings.^5^

Currently available glucose test strip enzymes include glucose oxidase, glucose dehydrogenase (GDH) nicotinamide dinucleotide, GDH flavin adenine dinucleotide, and GDH pyrroloquinolinequinone (GDH-PQQ).^2^^,^^6^ Among all these enzymes, GDH-PQQ is not glucose specific because it catalyzes the oxidation of not only glucose but also other sugars like maltose, galactose, and xylose.^5^ Vitamin C is a strong antioxidant that inactivates free radicals and can be oxidized on the surface of electrochemical strips to produce electrons and thereby increase current, which is detected as glucose by the GDH-PQQ strips. Isodextrin, an osmotic agent in peritoneal dialysate solution, is also metabolized in systemic circulation into various glucose polymers, mainly maltose and, hence, can be a source of error in POC-BG readings.^7^

Accu-Chek inform II is a commonly used device in the ICU as well as in non-ICU hospital settings, and it utilizes GDH-PQQ enzymatic activity in its mechanism of action for detecting BG.^1^ We also use Accu-Chek inform II devices on all our patients at our hospital. A review of adverse events (AE) associated with false glucose readings measured by GDH-PQQ-based glucose strips revealed that 56% of the reported cases were associated with severe clinical outcomes, 20% of which resulted in death. Agents most-commonly associated with AE were isodextrin-containing peritoneal dialysate solutions and maltose-containing intravenous immunoglobulin. Further, 17% of the reported cases occurred in outpatient settings, which is not entirely unexpected as peritoneal dialysis is mostly administered at home and intravenous immunoglobulin infusions can be administered in a provider’s office. It is important to note that the AE summarized in the report represents a small fraction because of under-reporting of AE in the database.^6^ Furthermore, the search was limited to codes and key words that did not include vitamin C; therefore, a considerable number of cases due to vitamin C interference might have been missed.^6^ If interference from vitamin C and other agents is unaccounted for or not determined at the time of POC-BG monitoring with these devices, providers may use excessive doses of insulin, resulting in hypoglycemic events with potential catastrophic consequences.^1^

The persistent effects of vitamin C infusion on POC glucose monitoring, even after it is stopped, is another concern. The timeline for interference has not been established and is very unpredictable, ranging from few hours to as long as 24 hours after the infusion has been stopped.^1^ In our case, the discrepancy persisted for at least 48 hours (24 hours after admission, ie, about 48 hours after the vitamin C infusion was stopped).

IV vitamin C interferes with GDH-PQQ test strips, and patients have demonstrated falsely-elevated POC-BG values during and/or immediately after the infusion period with discrepancies ranging from 10 to 200 mg/dL.^1^ Of note, this discrepancy is beyond the range of expected variability mentioned in standard guidelines.^8^ In 2016, the US FDA published its final guideline for POC-BG accuracy, which requires that 95% of all glucose values be within ±12% of results from a comparator method of glucose concentration and 98% of readings to be within ±15% of the comparator method. To date, none of the POC-blood glucose meters (BGM) have met standards according to FDA guidelines and maximal achievable accuracy was 85.4% to 86.5% of the comparable method. In 2018, FDA cleared the first POC-BGM for blood glucose monitoring in all hospital patients, specifically those receiving intensive medical intervention therapy. This device is the Nova Biomedical StatStrip Glucose Hospital Meter System. The use of every other POC-BG meter in the critical care setting is considered off label.^8^ A recent quality improvement project published by the Mayo Clinic compared 3 strip-based glucose meters (Roche Accu-Chek Inform II, Abbott Precision Exceed Pro, and Nova StatStrip). Of these, only the Nova StatStrip glucose meter specifically gave an “error” result for glucose readings that prompted clinicians to confirm POC-BG values with a venous blood sample.^9^

There are various sources of error in measuring POC-BG and these can pose a safety hazard to patient care. Chemical interference with the BG meter strip is only one of these. Other sources include operator errors, expired reagents in strips, environmental factors including temperature, humidity, and high altitude, and patient factors like hypoxia, dehydration, hemodynamic instability, hematocrit, hypertriglyceridemia, bilirubin, and uric acid levels.^5^ Physicians and nurses need to be aware of these potential sources of error while interpreting the results, and a thorough history, physical exam, and clinical state of the patient should be taken into account if POC and venous BG readings show contradictory results.

It is likely that, in the short term, capillary samples will be replaced by arterial/venous sampling methods that are analyzed by devices with higher accuracy than current POC-BG meters but still provide timely results using a smaller volume of blood than current laboratory methods. Continuous glucose monitors offer the possibility of more frequent BG monitoring in ICU patients through sensors placed in central lines or in the interstitial space.^6^

As most US-based institutions, including ours, are still utilizing POC glucose monitoring in ICU settings to monitor BG, it is worth considering using POC test strips that use enzymes with higher glucose specificity. The most common hand-held glucometers use an enzymatic oxidative reaction to produce an electron flow and, thus, generate current. The current generated is geometrically proportional to glucose level.^1^^,^^4^ Only glucometers that rely on this analytic method such as the Accu-Check Inform II models—known as amperometric detection—are susceptible to interference from vitamin C. Laboratory methods of detection, however, rely on spectrophotometric detection, which is independent of any current. Although vitamin C is present in the laboratory sample, there is no interference. Some hand-held POC glucose monitoring devices that use similar spectrophotometric methods are available but seem to be employed less frequently in critical care settings. These devices include the HemoCue (Mission Viejo) model of glucometers. Devices using amperometric detection but with in-built normalization algorithms for background interference, such as the Precision Xceed (Abbott Diabetes Care) and StatStrip (Nova Biomedical) models, can potentially reduce the effects of vitamin C on glucose values.^1^

We found no report of vitamin C causing apparent DKA as seen in our case. Our patient eventually died, and hypoglycemia probably contributed to the cause of death. We did not measure serum ascorbic acid levels in our patient.

## Conclusion

POC-BG monitoring is very commonly used in ICU settings to monitor BG as it is minimally invasive, convenient, and quick. However, physicians and nurses need to be aware that there are substances that can potentially interfere and alter POC-BG levels causing pseudohyperglycemia or pseudohypoglycemia. This can lead to catastrophic consequences and may result in increased morbidity and mortality in ICU settings. Whenever POC-BG is not consistent with laboratory values, the latter should be considered as true glucose values. FDA advises against the use of POC-BG meters in critical settings,^5^ and they should never be used to diagnose DKA. According to latest FDA guidelines, the only POC-BG meter approved for POC capillary testing in intensive care setting is the Nova Biomedical StatStrip Glucose Hospital Meter System.^8^ Furthermore, a detailed history regarding use of any interfering substances may provide a clue to diagnosis in the setting of contradictory glucose levels. The role of vitamin C in patients with burns and sepsis needs to be further explored.^3^

## Disclosure

The authors have no multiplicity of interest to disclose.
